# Highly sensitive hydrogen sensor based on graphite-InP or graphite-GaN Schottky barrier with electrophoretically deposited Pd nanoparticles

**DOI:** 10.1186/1556-276X-6-490

**Published:** 2011-08-10

**Authors:** Karel Zdansky

**Affiliations:** 1 Institute of Photonics and Electronics, Academy of Sciences of the Czech Republic, Chaberska 57, 18251 Prague 8, Czech Republic

**Keywords:** hydrogen sensor, metal nanoparticles, electrophoresis, Schottky barrier, InP, GaN

## Abstract

Depositions on surfaces of semiconductor wafers of InP and GaN were performed from isooctane colloid solutions of palladium (Pd) nanoparticles (NPs) in AOT reverse micelles. Pd NPs in evaporated colloid and in layers deposited electrophoretically were monitored by SEM. Diodes were prepared by making Schottky contacts with colloidal graphite on semiconductor surfaces previously deposited with Pd NPs and ohmic contacts on blank surfaces. Forward and reverse current-voltage characteristics of the diodes showed high rectification ratio and high Schottky barrier heights, giving evidence of very small Fermi level pinning. A large increase of current was observed after exposing diodes to flow of gas blend hydrogen in nitrogen. Current change ratio about 700,000 with 0.1% hydrogen blend was achieved, which is more than two orders-of-magnitude improvement over the best result reported previously. Hydrogen detection limit of the diodes was estimated at 1 ppm H_2_/N_2_. The diodes, besides this extremely high sensitivity, have been temporally stable and of inexpensive production. Relatively more expensive GaN diodes have potential for functionality at high temperatures.

## Introduction

Hydrogen gas (H_2_) monitoring sensors are in demand mainly for detection of H_2 _leakage in many industry productions such as, H_2 _filling stations, cryogenic cooling, research labs, etc. The gas is odorless, colorless, and highly inflammable, and therefore, effective H_2 _sensors are of great need for safety reasons. Highly sensitive and selective (i.e., exclusive to one gas) H_2 _sensors are needed in forming gas leak detectors for testing leaks in various equipment like vacuum apparatuses, refrigerators, heat exchangers or fuel systems in cars, etc. Such detectors contain highly sensitive H_2 _sensors and forming gas (noncombustive mixture of H_2 _in nitrogen) in place of expensive helium (the price of helium has recently risen sharply due to increased demand and limited resources) [1].

Thus, research on new H_2 _sensors has been well stimulated. Sensors based on semiconductor Schottky barriers principally exceed in sensitivity over the best results reported by sensors based on other sensing principles. The advantages of these sensors are also long life, low cost, and easy large-scale production. Palladium (Pd)/Si H_2 _sensors with two to three orders-of-magnitude change in current for 150 ppm of H_2 _in nitrogen (N_2_) were published already in 1981 [2]. About twice higher in sensitivity has been achieved with Pd/InP using electrophoretic deposition of Pd [3]. High sensitivity with about six orders-of-magnitude response to 5,000 ppm H_2 _in N_2 _has been achieved with porous Pd/GaN Schottky sensors [4]. It has been shown on the Pd/Si Schottky sensor that it responds linearly to H_2 _concentration in the range of three orders-of-magnitude, while the response starts to saturate above 1% of H_2 _in N_2 _and decreases faster below 10 ppm [5]. Similar behavior can be expected at other Schottky barrier sensors as well.

Nanoparticles of palladium or platinum are suitable for making H_2 _sensors based on Schottky barriers, intended to operate at room temperature. The reason is in the catalytic affectivity of these metal nanoparticles for dissociation of H_2 _molecules on metal-semiconductor interface. Ionized hydrogen atoms (protons), can form electric interface double layer with free electrons in the semiconductor, changing the height of the barrier which strongly affects barrier's electric properties. It has been shown that Pd/InP H_2 _sensors made by electrophoretic deposition of Pd nanoparticles are more sensitive than those made by thermal evaporation of Pd or even by electroless plating [3].

Recently, it was shown in our lab that the best H_2 _sensitivity of InP- or GaN-based structures could be achieved by combining electrophoresis of Pd nanoparticles with mechanical deposition of colloidal graphite for making Schottky contacts [6]. In this letter, the author reports on further studies of these structures.

## Experimental

Colloid solutions of Pd nanoparticles (NPs) in isooctane were prepared by reverse micelle technique with surfactant of sodium bis-(2-ethylhexyl) sulfoccinate (AOT) from water solutions of palladium chloride (PdCl_2_) and reducing agent hydrazine [7]. Shapes of Pd NPs in the colloid solution were monitored by a transmission electron microscope and/or by a scanning electron microscope (SEM). The Pd NPs were spherical, 7 nm in diameter, with 10% dispersion. Optical absorption peak due to surface-plasmon-resonance of Pd NPs in isooctane at 280 nm wave length was monitored by a split-beam photospectrometer. Pure chemicals and polished n-type InP and GaN (doping levels 2.5 × 10^15 ^and 2 × 10^17 ^cm^-1^) crystal wafers were purchased from recognized commercial companies as stated previously [6]. Each crystal wafer of 10 × 10 mm^2 ^size was first shortly treated in boiled methanol and then the unpolished side was procured with the all-area ohmic contact at room temperature by rubbing liquid solution of tin in gallium with a tin rod and a cotton-wool swab. Electrophoretic depositions (EPD) of Pd NPs onto polished InP or GaN were performed from the colloid solution by applying an electric field of 2,000 V/cm for 50 ms, with a 100-ms period for sufficient time. The field was held with the negative pole on the semiconductor wafer (sample) and the positive pole on the plane-parallel graphite electrode built in the tightly closed teflon cell [8]. The deposited layers on InP and GaN crystal wafers with Pd NPs were observed by SEM.

Schottky contacts were provided on the polished sides of the wafers (Pd NPs deposited or not deposited) by painting droplets of colloidal graphite in separate spots using a soft teflon needlepoint. The contacts were photographed on an optical microscope with Nomarski contrast and the photos were converted to the digital form for estimating contact areas. For that purpose, the digitized photos were modified to get the image with black background and white contact area, converted to the matrix form, and the contact area was integrated using a program on a computer.

Each Schottky contact and the all area ohmic contact on the other side of the wafer formed a rectifying Schottky diode. For measuring electrical properties, the diode was placed with the ohmic contact on a conducting platform, and a golden needlepoint on a bonze spring was touched on the Schottky contact, in the measuring cell. The cell was constructed with two holes to enable gas through-flow with free outlet to ambience for measuring gas sensitivity of electronic devices like H_2 _sensors.

## Results

**Figure 1:**
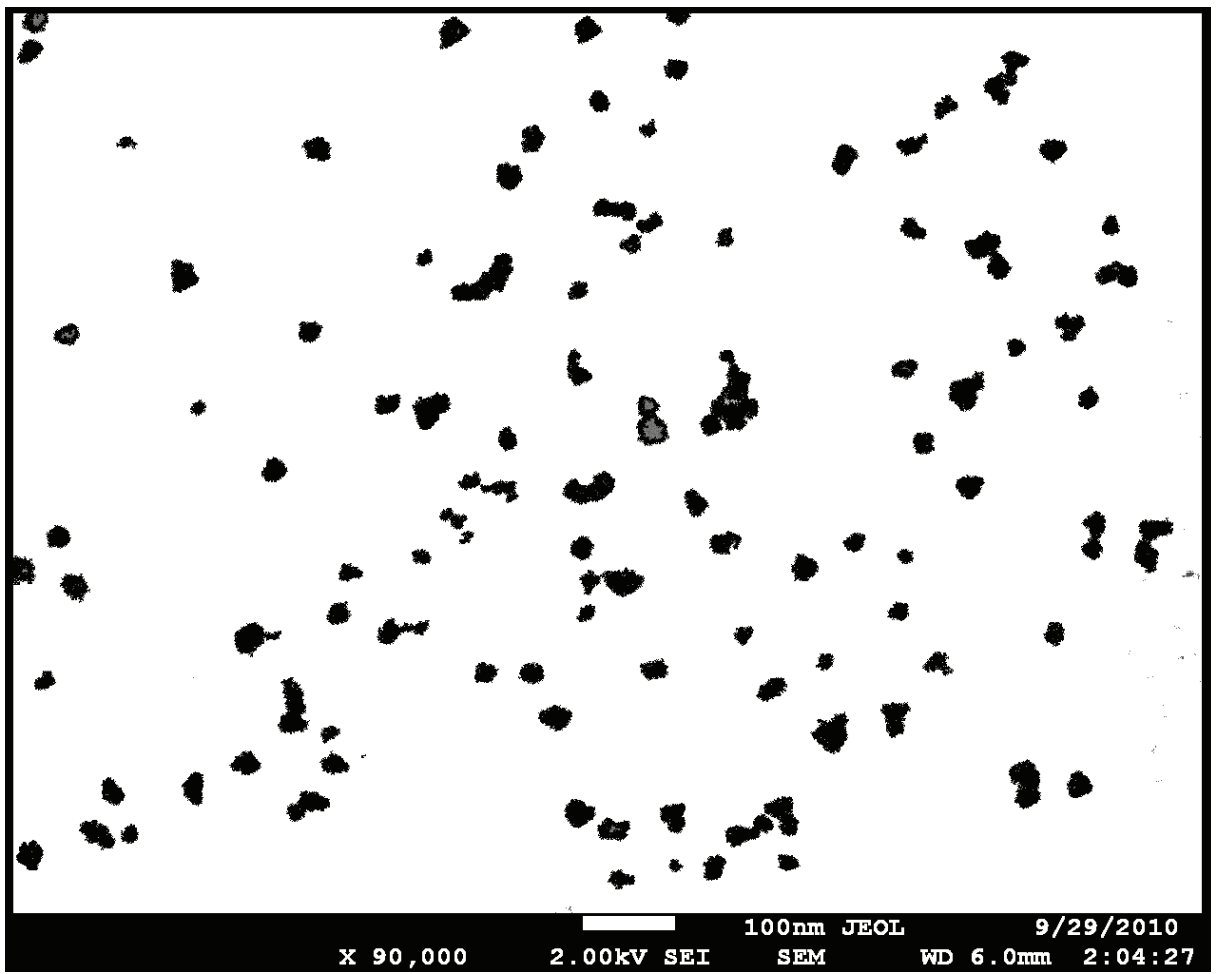
**SEM image of GaN after 2.5 h of electrophoretic deposition of Pd NPs**. The image was additionally processed to enhance the contrast. The scale 100 nm is shown with the bright bar at the bottom of the image.

**Figure 2:**
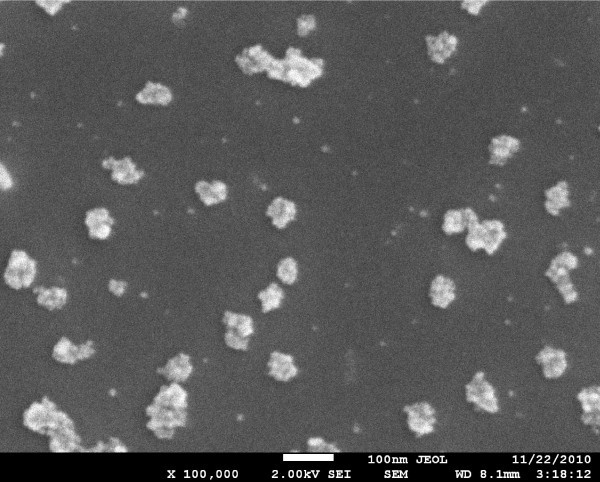
**SEM image of InP after 2 h of electrophoretic deposition of Pd NPs**. The scale 100 nm is shown with the bright bar at the bottom of the image.

The SEM image of GaN surface after EPD of Pd NPs can be seen in Figure 1. Rounded black spots represent Pd NPs; most of them are circular of about 10 nm in diameter and the others are their aggregations of various sizes. The image area is covered by the particles to about 10% only. The SEM image of InP surface deposited with Pd NPs from the same colloid solution following the same EPD process as in the previous case can be seen in Figure 2. In this case, most of the spots represent aggregated Pd NPs consisting of about ten spherical NPs of 10 nm in diameter. A similar image, but without aggregates, was obtained when a dried droplet of the colloid solution was observed on a copper grid coated with graphite. However, in such an image (not shown), there were no aggregates seen, despite that the colloid solution had been prepared several months earlier. It shows that aggregates of Pd NPs seen in Figures 1 or 2 did not arise in the colloid solution during storage, but they were created by the EPD process itself. A tendency to create aggregates was stronger in the case of EPD on InP than on GaN, as it can be seen by comparing Figure 2 with Figure 1.

**Figure 3:**
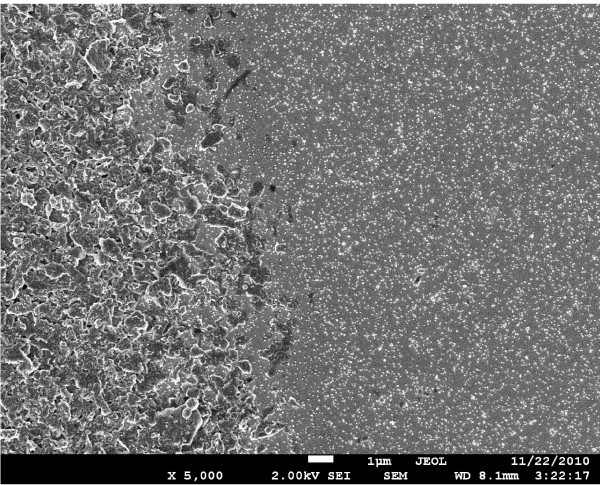
**SEM image of InP**. After 2 h of electrophoretic deposition of Pd NPs (right side) and graphite Schottky contact (left side). The scale 1 μm is shown with the bright bar at the bottom of the image.

**Figure 4:**
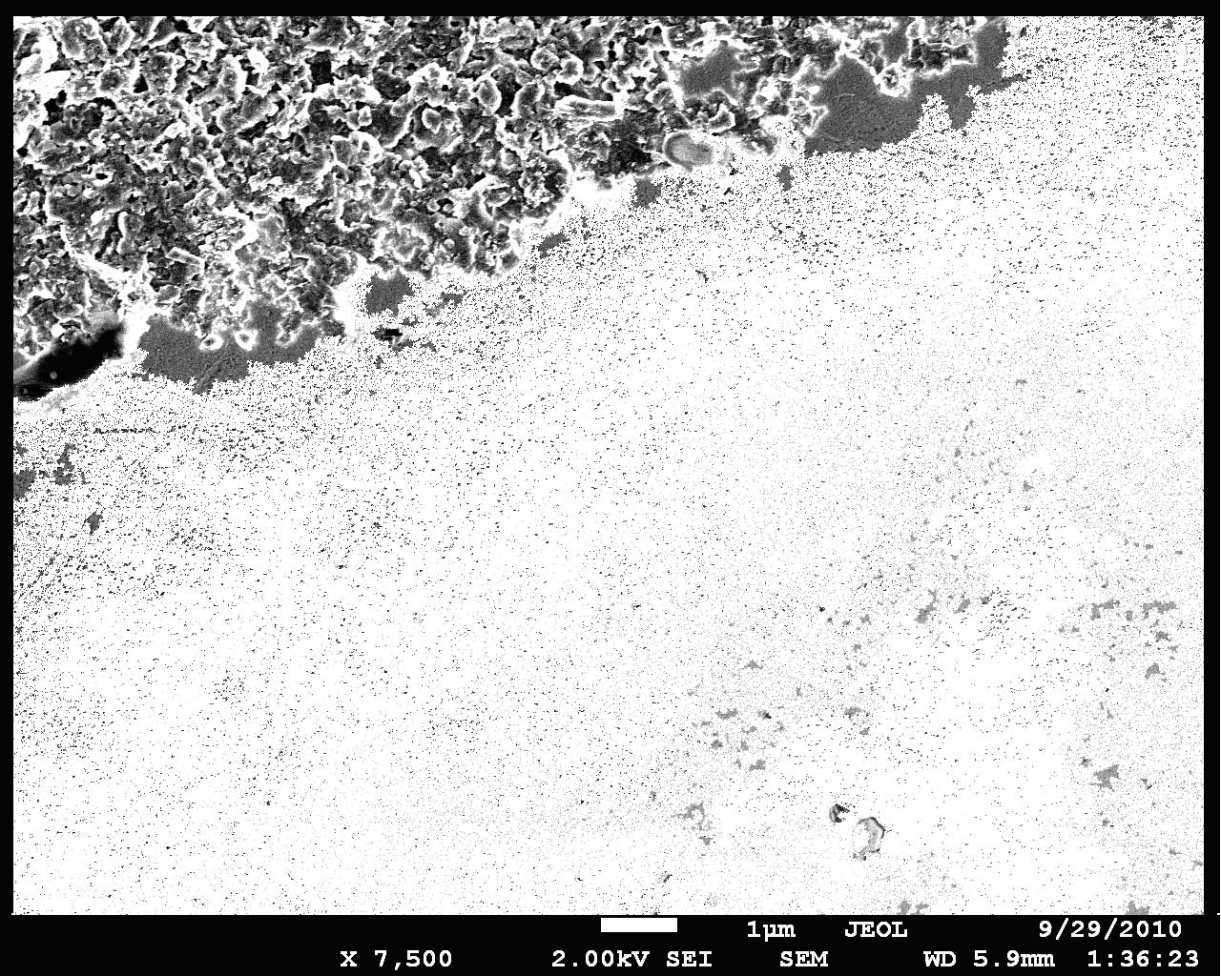
**SEM image of GaN**. After 2 h of electrophoretic deposition of Pd NPs (lower side) and graphite Schottky contact (left upper corner). The image was additionally processed to enhance the contrast. The scale 1 μm is shown with the bright bar at the bottom of the image.

The SEM image of a dried droplet of colloidal graphite, forming a Schottky contact on InP, can be seen on the left side of Figure 3. It is seen that the graphite layer consists of irregular particles of dimensions of 1 μm order-of-magnitude. A cognate image of graphite contact on GaN can be seen in the left upper corner of Figure 4. Likewise in this image, graphite particles can be well seen but small Pd NPs in the lower part are less distinct due to the smaller size of single Pd NPs in GaN surface than the size of aggregated NPs in InP surface (Figure 3).

**Figure 5:**
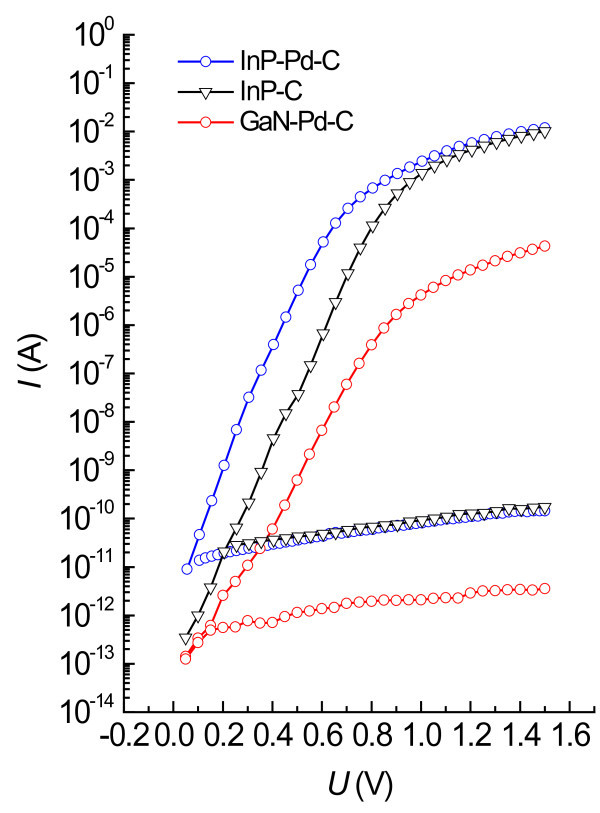
**Forward and reverse current-voltage characteristics of Schottky diodes**. Prepared by painting 0.0868, 0.0699, and 0.0769 mm^2 ^colloidal graphite on Pd NPs deposited InP (circles) and GaN (squares) and on plain InP (triangles).

Figure 5 Shows forward and reverse current-voltage characteristics of vertical diodes with Schottky contacts made by painting colloidal graphite on Pd NPs deposited surfaces of InP (InP-Pd-C) and GaN (GaN-Pd-C) and whole area ohmic contact on the opposite surface. Besides, there are also seen characteristics of diodes with graphite Schottky contacts made on the plain InP surface (InP-C). The areas of the Schottky contacts, estimated from photographs taken on the optical microscope, were 0.0868, 0.0699, and 0.0769 mm^2 ^for InP-Pd-C, GaN-Pd-C, and InP-C diode, respectively. It can be seen in Figure 5 that all diodes indicate a high rectification ratio of more than seven orders-of-magnitude. Notice that plain graphite diodes give (due to smaller leakage) smaller currents at low voltages than diodes with Pd NPs. Leakage currents of GaN-based diodes are about two orders-of-magnitude smaller than leakage currents of InP-based diodes.

All forward current-voltage characteristics show distinct linear parts in the semi-log scale in Figure 5. Using these linear parts, the Schottky barrier heights and ideality factors (IF) were evaluated as described in Ref. [7]. The height value was 0.873 eV and the ideality factor was 1.08 for InP-Pd-C diode, giving evidence that the thermionic emission primarily governed the electron transport in this case. In the case of GaN-Pd-C diode, the IF exited at 1.74 showing that a generation-recombination current (IF = 2) added to the thermionic emission current (IF = 1). When the linear part of the current-voltage curve of GaN-Pd-C diode was fitted with both currents added, the barrier height was estimated at 1.14 eV. The values of Richardson constants used in the evaluation were 9.24 and 26.4 A/(K·cm)^2 ^for InP and GaN, respectively.

**Figure 6:**
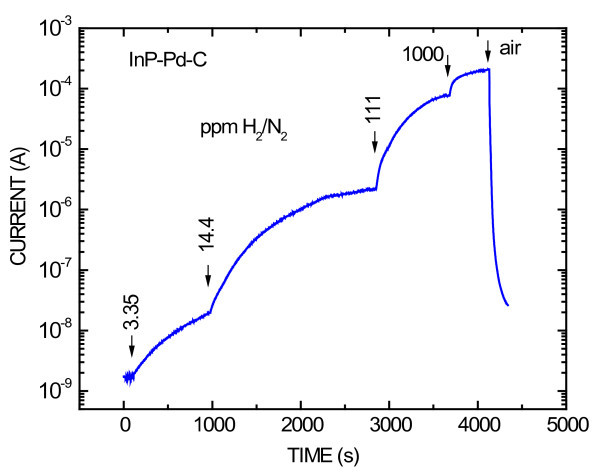
**Current transient responses of the InP-Pd-C Schottky diode**. Upon alternating exposure to the flow of gas blends H_2_/N_2 _and air. Transients upon exposure to four various gas blends are shown. The concentrations in parts per million are indicated with arrows. The diode was forward biased with the constant voltage of 0.1 V

Figure 6 shows current transient responses of the diode InP-Pd-C upon alternating exposure to the flow of various gas blends H_2_/N_2 _and air. The measurements started with the flow of air which showed virtually no change of current in comparison with that without the flow. Flows of four gas blends H_2_/N_2 _from 1,000 to 3 ppm were applied. The length of each flow was chosen to reach a stationary state when virtually no change of current was observed. It should be pointed that in the stationary state, the current did not change when the speed of flow was changed. The ratio of the current in the H_2_/N_2 _ambient to the current in the air ambient was 7 × 10^5 ^in the case of 0.1% H_2_/N_2_.

**Figure 7:**
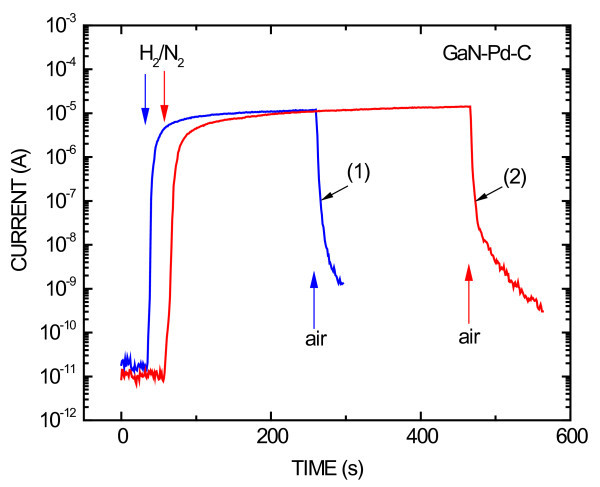
**Current transient responses of the GaN-Pd-C Schottky diode**. Upon alternating exposure to the flow of the gas blend 0.1% H_2_/N_2 _and of the air. Transients measured shortly after preparing the diode (1) and later after 3 months (2) are shown The diode was forward biased with the constant voltage of 0.5 V

Figure 7 shows current transient responses of the diode GaN-Pd-C upon alternating exposure to the flow of the gas blend 0.1% H_2_/N_2 _and of the air. There are two time developments in Figure 7: (1) measured shortly after preparing a diode and (2) measured lately, after 3 months' time. Characteristics of the two developments were the same, showing on a good time stability of the diode in this range of time. The ratio of the current in the H_2_/N_2 _ambient to the current in the air ambient was 7 × 10^5 ^in both cases. Also, the response time (change from air to H_2_/N_2 _exposure) and the recovery time (change from H_2_/N_2 _to air exposure) did not change after a 3-month history of the diode.

The diode InP-C, made by graphite on the plain InP, was also tested on the hydrogen sensitivity. However, there was no change of current when such voltage-biased diode was exposed to a gas containing hydrogen.

**Figure 8:**
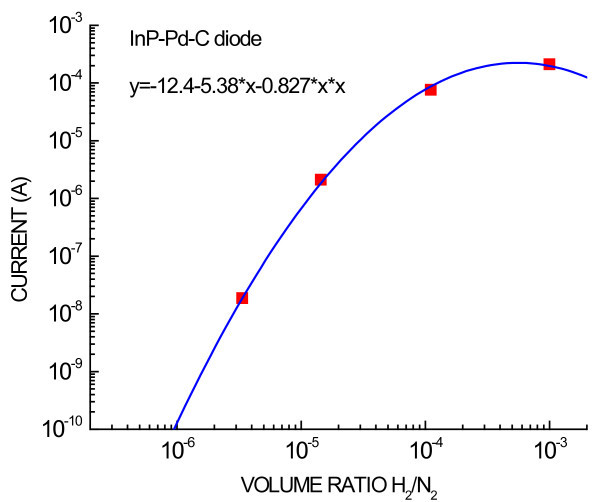
**Dependence of the current of InP-Pd-C diode on the concentration of H_2_/N_2_**. Four square points indicate values of current measured after long time exposure to the respective H_2_/N_2 _ratio (indicated in Figure 6). The full line is parabola in the log-log scale defined by the equation printed in the insert.

Four measured values of the current of InP-Pd-C diode were plotted in dependence on the concentration of H_2_/N_2 _in log-log scale as can be seen in Figure 8. The four plotted points can be well approximated with a parabolic curve. By the extension of this curve to lower concentrations, the hydrogen detection limit of the InP-Pd-C diode was estimated at 1 ppm H_2_/N_2_.

## Discussion

The Schottky diodes obtained by application of colloidal graphite on n-type InP and n-type GaN, marked InP-C, InP-Pd-C, and GaN-Pd-C are inexpensive but of very high quality, having low reverse leakage currents and high rectification ratios. Schottky barrier heights of 0.873 and 1.14 eV of InP-Pd-C and GaN-Pd-C diodes are much higher than those obtained by other methods, like thermal evaporation, which was, e.g., 0.55 eV in the case of Pd onto InP [9]. It shows on a very small or virtually negligible Fermi level pinning in these diodes so that any change in the Schottky barrier height should be equal to the change of the work function caused by an external charge appearing at the interface. Indeed, the measured values of Schottky barrier heights of 0.873 or 1.14 eV are close to differences between the electron work function of palladium metal 5.12 eV [10] and the electron affinity of InP, 4.38 eV [11] (0.74 eV) or of GaN, 4.1 eV [12] (1.02 eV). In principal, Fermi level pinning is caused by interface states in a semiconductor near the intimate contact with the metal. There are two basic ways creating interface states, physical breakings [9] and chemical reactions [13]. I believe that elimination of chemical reactions, due to forming Schottky barriers with colloidal graphite and surfactant wrapped Pd NPs, is the main reason for the absence of Fermi level pinning in the prepared diodes.

Only with small Fermi level pinning can Schottky diodes form effective gas sensors. Hydrogen sensing mechanism works as follows. H_2 _molecules penetrate through the porous graphite contact to the surface of the semiconductor where they are dissociated to hydrogen atoms due to the catalytic effect of the present Pd NPs. Positively charged hydrogen atoms (protons) after dissociation are attracted by electrons in the n-type semiconductor and form dynamically changing electric double layer. This electric double layer decreases the work function of the Schottky contact material and, consequently, it decreases the Schottky barrier height and increases the current of the voltage-biased diode. There are several favorable factors explaining the high H_2 _sensitivity of the diodes. First factor is the high Schottky barrier height and low leakage current of the interface between the graphite and InP or GaN semiconductor. Second factor is surfactant wrapped Pd NPs in concentration just partly covering the semiconductor surface, which does not lead to a serious decrease of the Schottky barrier height formed by the graphite and simultaneously it is sufficient for dissociation of penetrated hydrogen molecules. Third factor is the porous state of the graphite contact which allows easy penetration of hydrogen molecules to the interface with the Schottky barrier. Due to the abovementioned favorable factors, the current change ratio of the prepared diodes after exposure to 0.1% H_2_/N_2 _was 7 × 10^5 ^which represents more than two orders-of-magnitude improvement over the best result reported previously by H_2 _nanosensors [5].

Both types of diodes, based on InP and GaN, show about the same response and recovery time developments. The recovery time development consists of two parts: faster, just after the change from H_2 _to air exposure and a slower tail consisting of about 10% of recovering current change at the end, caused probably by slow release of H_2 _from the crystal lattice of Pd NPs [6]. The slow release shows that H_2 _in the crystal lattice of Pd is chemically bound in the form of palladium hydride (PdH_x_) [11]. This notion is supported by our further observation that the recovery tail is suppressed in cognate diodes with Pt NPs in place of Pd ones, which is in agreement with known experimental observations of PdH_x _and no observation of PtH_x _[14].

The current of InP-based diodes is more than one order-of-magnitude larger than the current of GaN-based diodes (see Figures 6 and 7), but the ratio of current change due to exposure to H_2 _is about the same for both types of diodes. InP diodes are advantageous for H_2 _measurements at room temperature due to their lower cost and larger current needing less laborious electronics. Beyond, more expensive GaN diodes are predicted for H_2 _measurements at high temperatures.

It should be noted that besides approximately 7 nm also approximately 10 nm Pd NPs were used to fabricate hydrogen sensors, and no demonstrable difference in their sensitivity was observed. It is important for prospective applications that the diodes are temporally stable as it is seen on the curve (2) in Figure 7. Good temporal stability of both, current-voltage characteristics and current transient responses to H_2 _exposure, has been proven for InP-Pd-C and GaN-Pd-C diodes.

## Conclusions

The Schottky diodes were prepared on polished single crystals of n-type InP or n-type GaN by painting colloidal graphite on the surface previously partly deposited with Pd NPs. The Pd NPs were deposited by electrophoresis from colloid solutions in isooctane prepared by chemical reduction of Pd-salt water solution in reverse micelles. The Schottky diodes showed current-voltage characteristics of low leakage currents and large rectification ratios, and they were much sensitive to H_2 _exposure with more than two orders-of-magnitude improvement over the best result reported previously [5]. Hydrogen detection limit of reported diodes was estimated at 1 ppm H_2_/N_2_.

## Competing interests

The author declares that they have no competing interests.
